# The types of hepatic myofibroblasts contributing to liver fibrosis of different etiologies

**DOI:** 10.3389/fphar.2014.00167

**Published:** 2014-07-22

**Authors:** Jun Xu, Xiao Liu, Yukinori Koyama, Ping Wang, Tian Lan, In-Gyu Kim, In H. Kim, Hsiao-Yen Ma, Tatiana Kisseleva

**Affiliations:** School of Medicine, University of California at San DiegoLa Jolla, CA, USA

**Keywords:** liver fibrosis, myofibroblasts, hepatic stellate cells, portal fibroblast, fibrocyte, carbon tetrachloride

## Abstract

Liver fibrosis results from dysregulation of normal wound healing, inflammation, activation of myofibroblasts, and deposition of extracellular matrix (ECM). Chronic liver injury causes death of hepatocytes and formation of apoptotic bodies, which in turn, release factors that recruit inflammatory cells (neutrophils, monocytes, macrophages, and lymphocytes) to the injured liver. Hepatic macrophages (Kupffer cells) produce TGFβ1 and other inflammatory cytokines that activate Collagen Type I producing myofibroblasts, which are not present in the normal liver. Secretion of TGFβ1 and activation of myofibroblasts play a critical role in the pathogenesis of liver fibrosis of different etiologies. Although the composition of fibrogenic myofibroblasts varies dependent on etiology of liver injury, liver resident hepatic stellate cells and portal fibroblasts are the major source of myofibroblasts in fibrotic liver in both experimental models of liver fibrosis and in patients with liver disease. Several studies have demonstrated that hepatic fibrosis can reverse upon cessation of liver injury. Regression of liver fibrosis is accompanied by the disappearance of fibrogenic myofibroblasts followed by resorption of the fibrous scar. Myofibroblasts either apoptose or inactivate into a quiescent-like state (e.g., stop collagen production and partially restore expression of lipogenic genes). Resolution of liver fibrosis is associated with recruitment of macrophages that secrete matrix-degrading enzymes (matrix metalloproteinase, collagenases) and are responsible for fibrosis resolution. However, prolonged/repeated liver injury may cause irreversible crosslinking of ECM and formation of uncleavable collagen fibers. Advanced fibrosis progresses to cirrhosis and hepatocellular carcinoma. The current review will summarize the role and contribution of different cell types to populations of fibrogenic myofibroblasts in fibrotic liver.

## INTRODUCTION

### PATHOGENESIS OF LIVER FIBROSIS

Hepati fibrosis is the outcome of many chronic liver diseases, including cholestatic liver diseases [primary sclerosing cholangitis (PSC), primary biliary cirrhosis (PBC), and secondary biliary cirrhosis (SBC)], and hepatotoxic liver diseases [hepatitis B virus (HBV), hepatitis C virus (HCV), alcoholic liver disease (ALD), and non-alcoholic steatohepatitis (NASH); Bataller and Brenner, 2005]. It is characterized by extensive deposition of extracellular matrix (ECM), including collagen Type I (Bataller and Brenner, 2005; Kisseleva and Brenner, 2007; Kisseleva and Brenner, 2008a,b, 2011). Hepatic fibrosis is caused by deregulation of physiological wound healing, and results in excessive production of ECM, mostly collagen type I, and scar formation. Activation of collagen producing myofibroblasts is critical for the pathogenesis of liver fibrosis (Bataller and Brenner, 2005; Kisseleva and Brenner, 2008b). Studies of animal models of liver fibrosis and patient material have demonstrated that activation of hepatic myofibroblasts plays a critical role in development of liver fibrosis. Increased number of myofibroblasts correlates with the severity of liver fibrosis in patients (Brenner et al., 2012). Therefore, myofibroblasts became an attractive target for anti-fibrotic therapy aimed to eliminate the source of activated hepatic myofibroblasts (Kisseleva et al., 2012; Liu et al., 2013). Myofibroblasts upregulate expression of α smooth muscle actin (α-SMA), non-muscle myosin, fibronectin, and exhibit stellate-like morphology. Myofibroblasts produce and secrete Collagen type I, the main component of the fibrous scar, and therefore, serve as a major source of ECM (Eyden, 2008; Watsky et al., 2010; Brenner et al., 2012). Although all myofibroblasts share common characterizations (such as expression of Col1α1, α-SMA, TGFβRI, TIMP1, PAI-1, activin, vimentin, fibronectin, activation of TGFβ1 signaling pathway), myofibroblasts may originate from distinct cellular sources, and the composition of myofibroblast population may vary dependent on the etiology of liver fibrosis. Identification of the cellular source(s) of fibrogenic myofibroblasts provides an invaluable insight into the pathophysiology of fibrogenic liver injury of different etiologies. Based on extensive studies, three cellular populations mainly contribute to hepatic myofibroblasts in response to chronic liver injury: hepatic stellate cells (HSCs; Kisseleva and Brenner, 2006, 2007), portal fibroblasts (PFs; Dranoff and Wells, 2010), and bone marrow (BM)-derived collagen producing cells (fibrocytes; Kisseleva et al., 2006; Scholten et al., 2011; Iwaisako et al., 2012; Kisseleva and Brenner, 2012). Activated HSCs (aHSCs) are the major source of myofibroblasts in chronic toxic liver injury (HBV, HCV infection, ALD, and NAFLD). PFs were implicated in injury caused by cholestic liver injury (primary and secondary biliary fibrosis; Bataller and Brenner, 2005). The role of fibrocytes in liver fibrosis remains unresolved. Recruitment of BM-derived fibrocytes into the injured liver was observed in animal models of both toxic and cholestatic liver fibrosis. However, contribution of fibrocytes to collagen producing myofibroblasts remains controversial. Identification of the origin of myofibroblasts plays a critical role in understanding the pathogenesis of liver fibrosis.

### CELL FATE MAPPING OF HEPATIC MYOFIBROBLASTS

Exploitation of Cre-LoxP systems in mice provided new opportunities for cell fate mapping and lineage tracing of myofibroblasts of different origins (Gilbertson, 2003; Kisseleva et al., 2012). This technique is based on cell-specific expression of Cre recombinase (overexpressed under cell/lineage-specific promoter) in mice. Upon crossing of Cre-expressing mice with reporter mice, such as Rosa26-flox-Stop-flox-YFP mice, in which flox-Stop-flox codon is knocked into Rosa26 locus, specific lineages of myofibroblasts and their progeny can be visualized and traced by expression of a fluorescent protein such as GFP or YFP.

## ORIGIN OF HEPATIC MYOFIBROBLASTS

Several sources of hepatic myofibroblasts have been identified (Kalluri and Neilson, 2003; Kisseleva and Brenner, 2006; Fallowfield et al., 2007; Gomperts and Strieter, 2007). HSCs and PFs are the liver resident mesenchymal cells (Asahina et al., 2009), they activate and proliferate in response to injury, and are believed to be the major source of myofibroblasts in fibrotic liver (Bataller and Brenner, 2005; Kisseleva et al., 2006, 2011; Parola et al., 2008; Roderfeld et al., 2009; Scholten et al., 2011). BM-derived fibrocytes and mesenchymal progenitor cells are recruited to the injured liver and become myofibroblasts (Bucala et al., 1994; Russo et al., 2006). Hepatic epithelial cells may undergo a transition into mesenchymal cells to contribute to the pathogenesis of liver fibrosis.

### HEPATIC STELLATE CELLS

Hepatic stellate cells are liver resident cells that originate from embryonic mesenchyme, and in adult healthy liver constitute approximately 10% of total liver cells. Under physiological conditions, HSCs exhibit a quiescent phenotype and serve as a major storage of Vitamin A. Quiescent HSCs (qHSCs) are located in the space of Disse (designated space between hepatocytes and sinusoidal endothelial cells), store retinoids in lipid droplets, and express desmin, neural markers, such as glial fibrillar associated protein (GFAP), synaptophisin, synemin, and nerve growth factor receptor p75 (Geerts, 2001; Bataller and Brenner, 2005). In addition, Nr1d2, Adipor1, Adpf, Dbp, Prei4, and Foxj1 were identified as unique markers associated with HSC quiescent phenotype (Liu et al., 2013). In response to fibrogenic liver injury and release of TGFβ1, qHSCs rapidly undergo activation. They downregulate Vitamin A-containing lipid droplets and neural markers, and differentiate into collagen Type I and αSMA-expressing aHSCs/myofibroblasts (Bataller and Brenner, 2005; Forbes and Parola, 2011). They also upregulate production of matrix metalloproteinases MMPs, especially MMP13, and their inhibitors TIMPs (Uchinami et al., 2006). Activation of HSCs is triggered mostly by increased hepatic levels of profibrogenic TGFβ1. In addition, PDGF, FGF, and CTGF also induce HSC activation (Pinzani and Marra, 2001; Reif et al., 2003; Hao et al., 2013). Recent studies have demonstrated that IL-6, leptin, and IL-17A can also trigger HSC activation (Bethanis and Theocharis, 2006; Michalopoulos, 2007; Meng et al., 2012). In addition aHSCs upregulate Crlf1, Spp1, Lox, LoxL2, IL-17Ra, Fosl1, and Folr1, genes that are uniquely associated with aHSC phenotype (Liu et al., 2013).

#### Transgenic mice to study HSCs

Reporter Collagen-α1(I)-GFP (Col-GFP) mice (Yata et al., 2003) are widely used to study activation of myofibroblasts in many types of organ fibrosis. Expression of GFP driven by Collagen-α1(I) promoter/enhancer (Yata et al., 2003) in these mice closely correlates with expression of Collagen Type I protein (Magness and Brenner, 1999; Magness et al., 2004), which is a major component of the fibrous scar (Bataller et al., 2003). These mice have been extensively characterized and have become a standard tool to visualize myofibroblasts contributing to fibrosis in liver, lungs, kidneys, and skin (Brenner et al., 1989; Stefanovic et al., 1997, 2000, 2002, 2005; Stefanovic and Brenner, 2003; Kisseleva et al., 2006, 2011; De Minicis et al., 2007; Lin et al., 2008; Taura et al., 2008, 2010; Österreicher et al., 2011; Inokuchi et al., 2011; Parsons et al., 2011; Scholten et al., 2011; Meng et al., 2012; Meurer et al., 2013). Using the Col-GFP mice we have recently demonstrated that aHSCs [isolated by flow cytometry as GFP^+^, Vitamin A^+^,and Desmin^+^ cells (Friedman et al., 1985; Bataller and Brenner, 2005; Seki et al., 2007)] comprise >92% of myofibroblasts in response to CCl_4_ (Kisseleva et al., 2012). Furthermore, significant progress has recently been made in the understanding of HSC biology based on HSC cell fate mapping. For this purpose, aHSCs were irreversibly labeled in CCl4-injured mice using Col1α1-Cre mice, Col1α2-Cre mice, and inducible Col1α1-ERCre mice. This technique allowed not only to trace and isolate aHSCs from a pool of non-parenchymal cells using flow cytometry-based cell sorting technique, but also to determine their cellular fate during regression of liver fibrosis (Kisseleva and Brenner, 2008a; Troeger et al., 2012). Hence, utilization of Cre expressing mice, in which Cre expression is driven by collagen Type I promoter, results in labeling of all hepatic myofibroblasts. Therefore, several successful attempts were made to generate HSC-specific Cre mice. Thus, vimentin-ERCre mice have recently been reported to label aHSCs (Troeger et al., 2012), while lecithin-retinol acyltransferase (Lrat)-ERCre mice induce tamoxifen-inducible Cre-LoxP recombination in HSCs independent of the status of their activation (Mederacke et al., 2013). Off note, previous attempts at HSC fate tracing have utilized hGFAP-Cre mice, however this yielded rather controversial results, since hepatic Cre-LoxP recombination in these mice generated three different phenotypes: recombination in HSCs (Kisseleva and Brenner, 2008a; Krizhanovsky et al., 2008; Lujambio et al., 2013), in cholangiocytes (Mederacke et al., 2013), or in both HSCs and cholangiocytes (Yang et al., 2008).

#### Reversibility of liver fibrosis

Many studies have clearly demonstrated that hepatic fibrosis is reversible in patients (e.g., HBV, HCV, biliary obstruction, or alcohol) and in experimental rodent models (alcohol feeding, CCl_4_, or bile duct ligation; Kisseleva and Brenner, 2011). Upon removal of the etiological source of the chronic injury, regression of liver fibrosis is associated with decreased cytokines and ECM production, increased collagenase activity, disappearance of myofibroblast population and dissolution of the fibrous scar (Iredale et al., 1998; Bataller and Brenner, 2005). Only recently has the fate of these myofibroblasts been revealed. The previous concept was that the myofibroblasts undergo apoptosis on the basis of documented senescence during reversal of fibrosis. We (Kisseleva et al., 2012) and subsequently others (Troeger et al., 2012) have used genetic marking to demonstrate an alternative pathway in which myofibroblasts revert to a quiescent-like phenotype in CCl_4_-induced liver injury and experimental ALD. Genetic marking of myofibroblasts enabled the quantitative mapping of the fate of these cells in experimental models of fibrosis and its reversal.

#### Inactivation of HSCs

The mechanism of HSC activation is well understood, but very little is known about HSC biology in the post-fibrosis phase. Using the Cre-Lox*P*-based genetic labeling of myofibroblasts, the fate of HSCs/myofibroblasts during recovery from CCl_4_-induced liver fibrosis has been elucidated, and it has been demonstrated that half of myofibroblasts undergo senescence (Krizhanovsky et al., 2008) and apoptosis (Iredale et al., 1998; Iredale, 2001), while half escape apoptosis during regression of liver fibrosis, downregulate fibrogenic genes and acquire a phenotype similar to, but distinct from, qHSCs (Kisseleva et al., 2012). In particularly, iHSCs more rapidly reactivate into myofibroblasts in response to fibrogenic stimuli and more effectively contribute to liver fibrosis. Inactivation of HSCs is associated with re-expression of lipogenic genes PPAR-γ, Insig1, and CREBP (She et al., 2005). Our findings in mice support the *in vitro* studies demonstrating the importance of PPAR-γ for the maintenance of quiescent phenotype (qHSCs; Gaca et al., 2003; She et al., 2005; Tsukamoto, 2005a,b; Wells, 2008). Following regression of liver fibrosis, HSCs restore their cellular mass and Vitamin A storage.

It remains unclear why some aHSCs apoptose during regression of liver fibrosis, and some aHSC inactivate (Kisseleva et al., 2012). Gene expression profiling of HSCs have demonstrated that those HSCs that survive and inactivate strongly upregulate Hsp1a/b heat shock proteins. Hsp1a/b proteins belong to Hsp70 family of heat shock proteins, but are expressed only in certain cell types in response to stress. We have demonstrated that Hsp1a/b knockout HSCs are more susceptible to glyotoxin or TNFa-induced apoptosis. In addition, regression of liver fibrosis in Hsp1a/b-/- is accompanied by massive apoptosis of aHSCs, suggesting that these two proteins may be involved in inactivation of HSCs (Kisseleva et al., 2012).

#### Epigenetic regulation

Understanding the mechanisms of HSC inactivation during regression of liver fibrosis is critical for finding new targets for anti-fibrotic therapy. Inactivation of HSCs is most likely regulated at an epigenetic level (versus genetic mutations; Mann et al., 2010; Tsukamoto et al., 2011). Epigenetics are heritable changes in gene function that occur without a change in the DNA sequence (Pepke et al., 2009). These changes, including nucleosome dynamics and histone modifications, cause structural alterations in the chromatin structure, and greatly affect gene expression. Post-translational modifications of the core histone subunits of nucleosomes are a fundamental mechanism by which the transcriptional activity of an associated gene locus can be regulated by methylation, acetylation, and phosphorylation (Henikoff, 2008; Park, 2009; Heinz and Glass, 2012). DNA methylation of genes expressed in qHSCs probably contributes to the maintenance of the quiescent phenotype. Upon activation, HSCs express DNA methyl-binding proteins (MeCP2) that promote silencing of antifibrogenic genes, such as IkB-α or PPAR-γ, and increase the expression of histone methyl transferase, leading in turn to enhanced transcription of collagen, TIMP-1, and TGF-β. PPARγ expression is associated with the adipogenic features of qHSC and must be silenced for the cell to activate into myofibroblasts (Hazra et al., 2004; She et al., 2005; Tsukamoto et al., 2012; Yang et al., 2012). A novel multi-step epigenetic network that controls activation of HSCs in the injured liver has recently been described, and involves activation of MeCP2 that causes alterations at the H3K27 methylation sites and generates transcriptionally repressed chromatin structure in the PPARγ promoter (She et al., 2005; Mann et al., 2010). Consistently, overexpression of PPARγ in aHSCs/myofibroblasts *in vitro* results in reversion of HSC activation, and reacquisition of their adipogenic characteristics (Hazra et al., 2004; Jaster et al., 2005).

#### The origin of HSCs

Hepatic stellate cells are believed to originate from mesenchymal cells during embryogenesis and recovery from liver injury (Cassiman et al., 2002; Bataller and Brenner, 2005). However, several important questions regarding HSC biology remain unknown. For example, what is the half-life of HSCs? What cell type(s) give rise to HSCs during liver organogenesis and in the adult liver? Finally, what triggers HSC proliferation during activation of liver fibrosis? HSCs share characterizations of both neural cells (expression of astrocyte marker GFAP, nestin and p75Ntr; Geerts, 2001), and mesodermal cells (smooth muscle marker desmin, adipocyte characterization, and ability to transdifferentiate into myofibroblasts). Cell fate mapping of Wnt+ cells (using Wnt1-Cre mice x Rosa-flox-Stop-flox-YFP mice) have demonstrated that HSCs do not originate from neural crest precursors which derive to all astrocytes (Cassiman et al., 2006). In turn, using lineage tracing of MesP1+ cells Asahina et al. (2011)have demonstrated that HSCs and PFs originate from mesenchymal precursors. In response to injury HSCs proliferate and activate into myofibroblasts. Cell fate mapping of aHSCs [Collagen-a1(I)-Cre mice x Rosa-flox-Stop-flox-YFP mice] have revealed that upon cessation of liver injury aHSCs either apoptose or inactivate into a quiescent-like state (Kisseleva et al., 2012). Interestingly, along with inactivated HSCs, which are genetically labeled and can be identified by YFP expression, we observed an emerging population of new HSCs without the “history” of collagen expression (Kisseleva et al., 2012). New studies are required to identify the source of HSCs in adult liver.

### PORTAL FIBROBLASTS

Portal fibroblasts reside underneath of the bile duct epithelium and are the major myofibroblast source in biliary cirrhosis, which is caused by biliary obstruction (Clouzeau-Girard et al., 2006; Dranoff and Wells, 2010). Biliary cirrhosis is characterized by dysregulated cholangiocyte proliferation and bile ductular enlargement (Jhandier et al., 2005). Although PFs are the first “responders” to liver injury caused by biliary obstruction (Wells et al., 2004), HSCs (and to lesser extend fibrocytes) also contribute to myofibroblast populations with the disease progression (Goddard et al., 1998).

#### Definition of PFs

Portal fibroblasts are a heterogeneous population and represent one of several fibroblast populations in the liver. The term “portal fibroblast” refers to any fibroblast in the portal region, and the term “portal myofibroblast” to any myofibroblast that originates in the portal area and is not derived from HSCs (Wells, 2014). Therefore, here we define aPFs as fibroblasts expressing fibulin 2, elastin, Thy1, interleukin 6, and the ectonucleotidase NTPDase 2, and do not express markers of stellate cells (Chapman and Eagles, 2007). There are other fibroblasts surrounding the central vein (second layer cells) and in the liver capsule (Bhunchet and Wake, 1992; Chapman and Eagles, 2007), many of which are derived from a common mesothelial or submesothelial progenitor cell that also gives rise to HSCs (Asahina et al., 2011). These populations have not been well defined or studied, and little is known about their contribution to fibrosis, epithelial maintenance, and other functions essential to the health of the liver and bile ducts.

#### Activation of PFs

Under physiological conditions, PFs comprise a small population of cells that surround the portal vein to maintain integrity of the portal tract (Dranoff and Wells, 2010). Activated PFs (aPFs) are implicated in the pathogenesis of cholestatic liver injury (Desmouliere et al., 1997), in which they proliferate and differentiate into α-SMA-expressing myofibroblasts that synthesize ECM (Desmouliere et al., 1997; Yata et al., 2003; Dranoff and Wells, 2010). The contribution of aPFs to liver fibrosis of different etiologies is not well understood, mainly because of the difficulties involved with the isolation of PFs and myofibroblasts. The most widely used method of aPF isolation from rat liver is based on enzymatic digestion followed by size selection (Wen et al., 2012). Cell outgrowth from dissected bile segments is still used to isolate rat PFs (Uchio et al., 2002), and after 10–14 days in culture PFs undergo progressive myofibroblastic differentiation (Kruglov et al., 2002). Unfortunately, this technique has several disadvantages since it requires multiple passaging and prolonged culturing which change the original phenotype of the cells (Dranoff and Wells, 2010). A more physiological method of studying PFs is precision-cut liver slices (PCLS), designed to maintain cell–cell and cell–matrix interactions and mimic the natural microenvironment of PFs, but this does not enable the study of purified populations of PFs. Only a few markers are available to identify PFs in the myofibroblast population, including gremlin, Thy1 (Knittel et al., 1999; Dudas et al., 2007; Yovchev et al., 2009), fibulin 2 (Knittel et al., 1999), IL-6, elastin (Goodpaster et al., 2008), the ecto-AT-Pase nucleoside triphosphate diphosphohydrolase-2 (NTPD2; Dranoff et al., 2002), and coffilin 1 (Bosselut et al., 2010). aPFs may also upregulate intermediate filament vimentin, the collagen receptor DDR2, and the calcium-binding protein S100A4 (Fsp1), and exhibit reactivity to the monoclonal antibody TE-7. In addition, the lack of desmin, cytoglobin, β_2_-macroglobulin, and Hand2, GFAP, p75^NGFr^, and Vitamin A distinguishes PFs from HSCs (Bataller and Brenner, 2005; Dranoff and Wells, 2010; Fausther and Dranoff, 2011). Identification of additional PF markers will advance our understanding of the pathogenesis of liver fibrosis.

#### Specific characteristics of aPFs

Two models of cholestatic liver injury are routinely used to study activation of PFs in mice. Bile duct ligation is caused by surgical obstruction of the common bile duct and mimics cholestatic liver disease in patients. Mice deficient in the canalicular phospholipid flippase (Mdr2/Abcb4^-/-^ mice) spontaneously develop liver injury (PSC; Shimamura et al., 2008), (Fichtner-Feigl et al., 2006; Jinnin et al., 2006; Fickert et al., 2009; Baghdasaryan et al., 2010; Mair et al., 2010; McHedlidze et al., 2013), which resembles PSC and most closely mimics MDR3 deficiency in patients,(Pope et al., 2005) which ranges from familial intrahepatic cholestasis to adult liver cirrhosis. The pathogenesis of liver injury in Mdr2^-/-^ mice is characterized by disruption of tight junctions and basement membranes of bile ducts, bile leakage into the portal tract, and formation of periportal biliary fibrosis (Jinnin et al., 2006; McHedlidze et al., 2013). aPFs were proposed to contribute to ECM deposition in Mdr2^-/-^ mice and in response to bile duct ligation (Jinnin et al., 2006; Fickert et al., 2009). In the case of cholestatic liver injury aPFs respond rapidly to increasing levels of TGF-β1 (Liu et al., 2003) by upregulation of Col-α1(I), α-SMA, TIMP1, TGF-β2 (Wells et al., 2004), PAI-1, elastin (Dranoff and Wells, 2010), fibronectin (Knittel et al., 1999; Ji et al., 2012), and of CD73 ecto-enzyme (Li et al., 2007; Fausther et al., 2012; Ji et al., 2012), and differentiate into collagen producing myofibroblasts. BDL-aPFs also secrete TGF-β1 and TGF- β2 (Wells et al., 2004). FGF-2 facilitates activation of Pfs as well (Venkataraman et al., 1999). Binding of FGF-2 to its tyrosine kinase receptors FGFRs, and subsequent activation of Ras-MEK-Erk1/2 signaling causes rapid proliferation and migration of aPFs (Itoh and Ohta, 2013). Proliferation and activation of aPFs was shown to be blocked by Curcumin, a non-steroidal yellow pigment found in rhizomes of the perennial herb Curcuma longa, which blocks ERK1/2 phosphorylation in aPFs, and thus has a potential to reduce cholestasis-induced fibrogenesis (Baghdasaryan et al., 2010). aPFs are believed to be the “first responders” to cholestatic liver injury which contribute to a significant number of collagen producing myofibroblasts. aPFs begin to proliferate immediately after bile duct ligation giving rise to a population of desmin^-^Vitamin A^-^ α-SMA^+^ myofibroblasts adjacent to proliferating bile ducts and connective tissue stroma (Wells, 2014). Activation of PFs proceeds activation and proliferation of HSCs, suggesting that activation of HSCs is secondary to PFs in response to cholestatic liver injury (Tuchweber et al., 1996; Desmouliere et al., 1997). In addition, PFs play a role in maintenance of the peribiliary stem cell niche, regulation of cholangiocyte proliferation, and deposition of specific matrix proteins, such as elastin and other components of microfibrils, providing stability to ducts and the vasculature under conditions of increased ductal pressure (Wells, 2014). Recent studies have also proposed that HSCs and PFs occupy different niches which yield myofibroblasts with specialized functions dependent on the etiology of liver injury. If the HSC niche is activated in response to hepatotoxic damage and hypoxia to produce myofibroblasts that mediate hepatocellular healing, then the portal fibroblast niche is activated by the ductular reaction to produce PF–derived myofibroblasts that regulate scar formation (Lemoinne et al., 2013; Wells, 2014).

#### Origin of portal fibroblast

Recent studies have suggested that during embryonic development PFs, HSCs and vascular smooth muscle cells (VSMC) originate from mesenchymal precursors of septum transversum (Suzuki et al., 2008; Asahina et al., 2009). Lineage tracing of precursor cells expressing mesodermal marker, MesP1^Cre^ providesevidence that septum transversum originates from mesoderm (Asahina et al., 2011). At day 9.5 (E9.5) of mouse embryogenesis, when the hepatic bile duct system and sinusoid start to form, septum transversum is invaded by foregut endoderm. At E12.5, the perihepatic membrane mesothelial cells and submesothelial cells give rise to the mesenchymal cell precursors which invade into the liver mesenchyme (Asahina et al., 2011; Rinkevich et al., 2012). Several cell markers of these mesenchymal cell precursors have been identified, including Mesothelin, Desmin, p75NTR, Wt1, and ALCAM. These mesenchymal precursors interact with endodermal hepatoblasts to induce the differentiation of hepatic parenchyma. It remains unclear at which time point and specific stage of embryonic development PF and HSC precursors diverge from each other and retain expression of cell specific markers throughout adulthood distinguishing these two cell types from each other. One explanation which has evolved is that positioning during hepatic lobule formation may designate functional specialization leading to divergence of PFs from HSCs. In concordance, fetal PFs secrete BMPs, Jagged1, and Hedgehog ligands to induce signaling critical for differentiation of hepatoblasts into cholangiocytes during bile canaliculi and lumen formation (Dranoff and Wells, 2010; Fabris and Strazzabosco, 2011), while fetal HSCs were implicated in supporting proliferation of hepatoblasts by producing HGF, pleiotrophin and FGF10 (Asahina et al., 2002; Berg et al., 2007).

### FIBROCYTES

#### Bone marrow derived collagen producing Fibrocytes

Fibrocytes were first described by Bucala et al. (1994), Bucala (2008), and Grieb et al. (2011) and are defined by the simultaneous expression of CD45 and collagen type I. Fibrocytes possess dual characteristics of fibroblasts (expression of collagen type I, fibronectin, and vimentin) and hematopoietic cells (CD45, CD34, MHCII, CD11b, Gr1, Ly6c, CD54, CD80, CD86, CCR2, CCR1, CCR7, CCR5; Abe et al., 2001; Quan et al., 2004). Under physiological conditions, fibrocytes have a spindle-like shape. In response to injury (including liver injury), or stimulation by TGF-β, fibrocytes downregulate expression of hematopoietic markers and rapidly differentiate into α-SMA^+^ myofibroblasts (Quan et al., 2004; Quan and Bucala, 2007). Due to their ability to give rise to fibrogenic myofibroblasts, fibrocytes were implicated in the pathogenesis of skin, lung, kidney, and liver fibrosis (Phillips et al., 2004; Moore et al., 2005, 2006; Kisseleva et al., 2006; Sakai et al., 2006; Ishida et al., 2007; Strieter et al., 2007; Wada et al., 2007; Lin et al., 2008; Grieb et al., 2011).

#### Approach to identify and study fibrocytes in mice

We have developed a new functional method to distinguish fibrocytes from liver resident fibrogenic myofibroblasts (Kisseleva et al., 2006; Higashiyama et al., 2009), or other BM-derived cells (Scholten et al., 2010; Kisseleva et al., 2011). Based on the ability of a collagen-α1(I)-GFP transgene (Yata et al., 2003) to drive GFP expression specifically in fibrocytes, but not other cells of the hematopoietic system [such as activated macrophages (Pilling et al., 2009)], fibrocyte-specific BM chimeric mice Col-GFP→wt mice (generated by transplantation of Col-GFP BM into lethally irradiated wild type recipient mice) became a useful tool to study fibrocyte biology. Liver injury causes rapid recruitment of BM-derived fibrocytes (Russo et al., 2006; Kallis and Forbes, 2009) to fibrotic livers of Col-GFP→wt mice. Using Col-GFP→wt mice, we have identified that fibrogenic liver injury activates several populations of fibrocytes, hepatic (Kisseleva et al., 2006), splenic (Kisseleva et al., 2011), and BM CD45^+^Col^+^ fibrocytes (Brenner and Kisseleva, 2011; Scholten et al., 2011).

#### Fibrocytes contribute to liver fibrosis

**FIGURE 1:**
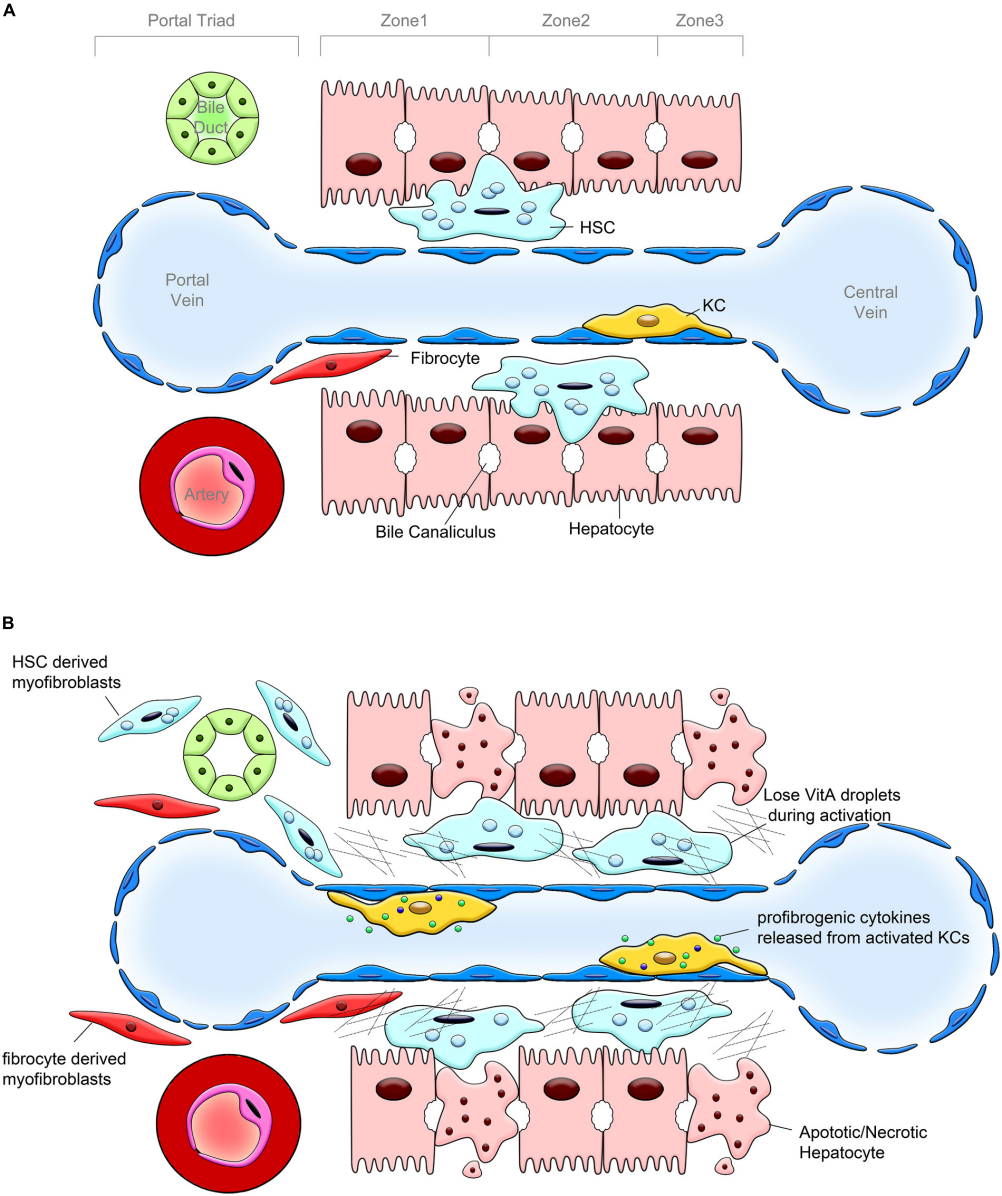
**Pathogenesis of liver fibrosis. (A)** Hepatic lobular structure under physiological conditions. Hepatic sinusoid, cholangiocytes and hepatocytes, endothelial cells, hepatic stellate cells (HSCs) and portal fibroblasts (PFs), and Kupffer cells (KCs) are the components of hepatic lobule. The bile duct, portal vein and hepatic artery form the portal triad. HSCs are located in the space between hepatocytes and sinusoidal endothelium, designated as the space of Disse. HSCs are considered as liver pericytes, they contain lipid droplets and serve as the major storage of vitamin A. KCs represent a population of hepatic macrophages. Only few fibrocytes are present in the healthy liver. **(B)** Changes in the hepatic lobule caused by chronic liver injury. In response to chronic liver injury, hepatocytes undergo apoptosis and release factors that recruit Kupffer cells, BM macrophages, and fibrocytes into the damaged liver. KCs, BM macrophages, and fibrocytes release TGFβ1, the most potent profibrogenic cytokine, which activates HSCs into collagen Type I expressing myofibroblasts. HSCs, PFs and, to a lesser extent fibrocytes, deposit extracellular matrix (ECM).

CD45^+^Col^+^ fibrocytes migrate to the injured liver in response to bile duct ligation (BDL, cholestatic injury) and comprise ≈4–6% of the collagen type I expressing cells (Kisseleva et al., 2006). Similarly, fibrocytes are recruited into CCl_4_-dameged liver (**Figures 1A,B**), where they can differentiate into α-SMA^+^ myofibroblasts *in vivo* (Scholten et al., 2011), suggesting that fibrocyte recruitment to the liver is a universal mechanism in the pathogenesis of liver fibrosis. In support, a significant number of fibrocytes is recruited in Abcb4 knockout mice, a genetic model of spontaneous liver fibrosis. There was a significant flux of fibrocytes to the liver in these mice, such that fibrocytes contributed ≈50% to the liver myofibroblast population (Roderfeld et al., 2009). Although the mechanism of fibrocyte recruitment caused by this genetic deficiency is not completely understood, it demonstrates that fibrocytes have a strong potential to differentiate into myofibroblasts under specific circumstances. For example, adoptively transferred fibrocytes that differentiate into collagen producing cells when cultured on plastic, exacerbate pulmonary fibrosis in mice (Phillips et al., 2004). This phenomenon emphasizes fibrocyte plasticity, fibrogenic potential, and organ-specific differentiation.

#### Migration of fibrocytes to fibrotic liver

Development of liver fibrosis is mediated by increased secretion of biologically active TGF-β1, intestinal lipopolysaccharide (LPS) and pro-inflammatory chemokines CCL2, CCL7, CCL12, and CCL3, which are the ligands for chemokine receptors CCR2 and CCR1 on hepatic and BM-derived cells (Seki et al., 2009a,b). TGF-β1 triggers fibrocyte mobilization into fibrotic liver, suggesting that regulation of fibrocyte migration by TGF-β1 might be a general characteristic of fibrogenic injury of the liver and other parenchymal organs (Phillips et al., 2004; Balmelli et al., 2007; Hong et al., 2007). Moreover, recruitment of fibrocytes from the BM to the injured liver is regulated by CCR2 (Scholten et al., 2010), a chemokine receptor that is also required for fibrocyte migration into fibrotic lungs and kidneys (Phillips et al., 2004; Moore et al., 2005; Sakai et al., 2006; Balmelli et al., 2007; Hong et al., 2007; Ekert et al., 2011). Unlike lungs and kidneys (Phillips et al., 2004; Moore et al., 2005, 2006; Sakai et al., 2006; Ishida et al., 2007; Wada et al., 2007), recruitment of fibrocytes to fibrotic liver was shown to be dependent on CCR1 (Scholten et al., 2010), indicating that differential expression of chemokine receptors may determine an organ-specific migration of fibrocytes in response to fibrogenic injury.

#### BM is the source of hepatic fibrocytes

Studies using bone marrow transplantation (BMT) in mice have established that BM is the source of fibrocytes. Under physiological conditions fibrocytes are primary located in the BM (comprise 0.1% of mononuclear cells), but proliferate and transmigrate with the blood stream in response to injury (Quan et al., 2004). Fibrocytes have been isolated from fibrotic tissues, spleens and peripheral blood (Bucala et al., 1994; Quan et al., 2004; Scholten et al., 2011). BM-derived fibrocytes have a spindle-like shape, but in response to TGF-β1, they rapidly obtain a myofibroblast phenotype (Quan et al., 2004). These BM fibrocytes possess all classical characteristic of fibrocytes: expression of collagen, CD45, CD11b CD34, Gr1, CD80, and CD86 (Quan et al., 2004; Bellini and Mattoli, 2007; Reilkoff et al., 2011). BM-derived fibrocytes migrate specifically into fibrotic liver when adoptively transferred into BDL mice (Scholten et al., 2011), supporting the notion that BM fibrocytes are a source of hepatic fibrocytes/myofibroblasts.

#### Development of liver injury is accompanied by recruitment of fibrocytes to the spleen

In addition to the injured organ, recruitment of CD45^+^Col^+^ fibrocytes to the spleen has been documented during development of liver (Kisseleva et al., 2006; Niedermeier et al., 2009) and kidney fibrosis (Sakai et al., 2006). Hepatotoxic injury (CCl_4_), TGF-β1, LPS, or infection with *Listeria monocytogenes (Lm)* trigger migration of fibrocytes from the BM to the spleen and liver (Kisseleva et al., 2011). Moreover, the spleen functions as a major reservoir of immature fibrocytes. Splenic CD45^+^Col^+^ fibrocytes express myeloid markers and resemble CD115^+^CD11b^+^ monocytes (Kisseleva et al., 2011). Migration of fibrocytes specifically to *Lm*-infected spleen and liver indicate their potential role in innate immunity. Although the biological significance of splenic fibrocytes is not understood, our recent study suggests that CD45^+^Col^+^ fibrocytes are capable of differentiating according to their microenvironment, giving rise to different subtypes of fibrocyte-like cells with distinct roles during tissue repair and fibrosis (Curnow et al., 2010). Consistent with this observation, splenic fibrocytes uniquely upregulate a variety of antimicrobial factors [myleoperoxidase, cathelicidin (mCRAMP), defensins; Kisseleva et al., 2011], and expression of MHC II (Scholten et al., 2011). Although splenic fibrocytes lack phagocytic activity, they have developed alternative mechanisms to combat infection. First, splenic fibrocytes confine bacterial spread at the site of infection by entrapment of bacteria in extracellular DNA-based structures (“DNA traps”; Kisseleva et al., 2011), a mechanism previously identified only in neutrophils, eosinophils, mast cells, and macrophages (Brinkmann et al., 2004; von Kockritz-Blickwede et al., 2008; Yousefi et al., 2008; Chow et al., 2010). Fibrocytes kill bacteria by secretion of cathelicidin into the DNA-based framework (Kisseleva et al., 2011). Second, upon migration to the spleen, fibrocytes strongly upregulate expression of MHC II (Scholten et al., 2011) and mediate adaptive immunity by presenting antigens to naïve T cells (Chesney et al., 1997; Balmelli et al., 2005) *in vivo* and *in vitro* (Kisseleva et al., 2011). Although the antimicrobial properties of fibrocytes are aimed at stopping infection (Brinkmann and Zychlinsky, 2007), release of nuclear DNA and lysosomal peptides into the extracellular space facilitates inflammation, and was shown to cause autoimmune vasculitis (Kessenbrock et al., 2009).

### EPITHELIAL MESENCHYMAL TRANSITION (EMT) AND LIVER FIBROSIS

The origin of myofibroblasts from hepatic epithelial cells is controversial. Although several evidence indicated that, under prolonged *in vitro* culturing, hepatocytes and cholangiocytes upregulate myofibroblast marker aSMA and suppress epithelial cellular marker (Kisseleva and Brenner, 2008a; Choi and Diehl, 2009; Kalluri, 2009), the fate mapping-based studies have clearly demonstrated that hepatocytes and cholangiocytes, or their precursors do not undergo EMT in response to experimental models of liver fibrosis and do not give rise to myofibroblasts (Scholten et al., 2010; Chu et al., 2011; Österreicher et al., 2011). These studies have shown that genetic labeling of hepatocytes (using Albumin-Cre mice), cholangiocytes [using cytokeratin 19 (K-19)-Cre mice] and their precursors did not yield generation of myofibroblasts *in vivo*. Furthermore, expression of S100A4 (Fsp1), a marker which is associated with EMT progression, and widely used to detect cells undergoing EMT, has been found to be expressed not only by subsets of fibroblasts but also by myeloid cells, suggesting that the role of this protein in the pathogenesis of liver fibrosis has to be reevaluated.

## HUMAN LIVER DISEASES AND FIBROGENIC CELL POPULATIONS

It is well established that liver myofibroblasts are the primary effector cells responsible for the extensive ECM accumulation and scar formation observed during hepatic fibrosis (Fausther et al., 2013). Liver myofibroblasts represent the critical targets for antifibrotic therapies, and therefore, it is important to determine the composition and contribution of distinct subsets of myofibroblasts to clinical and experimental liver fibrosis of different etiologies (Fausther et al., 2013). Based on the analysis of liver material from patients with advanced fibrosis and cirrhosis, three distinct mesenchymal myofibroblast-like liver cell subpopulations can be discerned: portal/septal myofibroblasts, interface myofibroblasts and perisinusoidally located aHSCs. Septal myofibroblasts share more characteristics with portal myofibroblasts than with HSCs perhaps suggesting their common descent (Cassiman et al., 2002). However, contribution of these subpopulations vary dependent on underlying etiology of liver fibrosis. Thus, primarily activation of HSCs with upregulation of α-SMA is observed in patients with viral hepatitis B and C (Giannelli and Antonaci, 2005; Friedman, 2008a,b). In addition, viral antigens and dsDNA are able to activate HSCs via CD4/CD8 lymphocyte mediated manner (Enzan et al., 1994; Muhanna et al., 2008). In cholestatic liver fibrosis, both aPFs and aHSCs contribute to the ECM deposition, and the major accumulation of collagen expressing cells have been observed around proliferating bile ducts (Goddard et al., 1998).

Recruitment of fibrocytes in patients with liver fibrosis has been documented. The number of circulating fibrocytes was shown to correlate with the severity of fibrosis induced by HCV (Bucala, 2008), lung fibrosis (Strieter et al., 2009) and Crohn’s disease (Sahebally et al., 2013), suggesting that fibrocytes may serve as a prognostic marker, and become a novel target for anti-fibrotic therapy in fibrosis. However, the function of fibrocytes in the liver is not well understood. Thus, fibrocytes were implicated to be a major source of collagen Type I producing cells in patients with nephrogenic systemic fibrosis (NSF; Cowper and Bucala, 2003; Galan et al., 2006). Meanwhile, recent reports suggest that fibrocyte function is not limited to ECM deposition, and fibrocytes also act to promote fibrosis via paracrine actions, such as secretion of growth factors, proteases, and matricellular proteins, which affect resident epithelial and mesenchymal cells towards pathologic remodeling (Kleaveland et al., 2014).

Although the data supporting the concept of EMT in adult patients with liver fibrosis has recently been critically questioned, there is evidence that EMT occurs during mammalian embryogenesis and cancerogenesis. Therefore, it is quite possible that pediatric patients who are born with biliary atresia may retain a rudimentary mechanism by which bile ducts undergoing EMT may account for prominent bile ductular proliferation and fibrogenesis (Deng et al., 2011). Liver sections from patients with biliary atresia were evaluated to detect antigen for the BECs marker 4 and cytokeratin-7 (CK-7), proteins (fibroblast-specific protein 1, also known S100A4; the collagen chaperone heat shock protein 47, HSP47) characteristically expressed by cells undergoing EMT, as well as myofibroblast marker a-smooth muscle actin (a-SMA).

## CONCLUSION

The composition of myofibroblasts varies dependent on the etiology of liver fibrosis. Hence, liver resident HSCs and portal/septal fibroblasts are considered to be the major source of fibrogenic myofibroblasts in the damaged liver and serve as primary targets for anti-fibrotic therapy. Specifically, recent studies on epigenetic regulation of hepatic myofibroblasts provide new opportunities for drug discovery (Mann and Mann, 2009; Mann et al., 2010). Furthermore, identification of inactivated phenotype in HSCs, suggests that aHSCs can be manipulated to stop producing collagen and reverse to their quiescent like state. Unfortunately, much less is known about PFs. Although activation of PFs is restricted mostly to cholestatic liver injury, new tools are required to study this population of mesenchymal cells contributing to liver fibrosis.

## Conflict of Interest Statement

The authors declare that the research was conducted in the absence of any commercial or financial relationships that could be construed as a potential conflict of interest.

## ABBREVIATIONS

α-SMA: α-smooth muscle actin
aHSCs: activated HSCs
BDL: bile duct ligation
BM: bone marrow
CCl_4_: carbon tetrachloride
ECM: extracellular matrix
EMT: epithelial-to-mesenchymal transition
HCC: hepatic cellular carcinoma
HSCs: hepatic stellate cells
iHSCs: inactivated HSCs
KC: Kupffer cells
LPS: lipopolysaccharide
PF: Portal fibroblast
qHSCs: quiescent HSCs.
